# Mexican *BRCA1* founder mutation: Shortening the gap in genetic assessment for hereditary breast and ovarian cancer patients

**DOI:** 10.1371/journal.pone.0222709

**Published:** 2019-09-23

**Authors:** Veronica Fragoso-Ontiveros, Jose Antonio Velázquez-Aragón, Paulina Maria Nuñez-Martínez, Maria de la Luz Mejía-Aguayo, Silvia Vidal-Millán, Abraham Pedroza-Torres, Yuliana Sánchez-Contreras, Miguel Angel Ramírez-Otero, Rodolfo Muñiz-Mendoza, Julieta Domínguez-Ortíz, Talia Wegman-Ostrosky, Juan Enrique Bargalló-Rocha, Dolores Gallardo-Rincón, Nancy Reynoso-Noveron, Cristian Arriaga-Canon, Abelardo Meneses-García, Luis Alonso Herrera-Montalvo, Rosa Maria Alvarez-Gomez

**Affiliations:** 1 Hereditary Cancer Clinic, National Cancer Institute, Mexico city, Mexico; 2 Molecular Biology Laboratory, National Paediatrics Institute, Mexico city, Mexico; 3 Research Direction, National Cancer Institute, Mexico city, Mexico; 4 Breast Cancer Department, National Cancer Institute, Mexico city, Mexico; 5 Medical Oncology Department, National Cancer Institute, Mexico city, Mexico; 6 Epidemiology Department, National Cancer Institute, Mexico city, Mexico; 7 General Direction, National Cancer Institute, Mexico city, Mexico; CNR, ITALY

## Abstract

The deletion of exons 9 to 12 of *BRCA1* (9–12 del *BRCA1*) is considered a founder mutation in the Mexican population. We evaluate the usefulness of the target detection of 9–12 del *BRCA1* as the first molecular diagnostic strategy in patients with Hereditary Breast and Ovarian Cancer (HBOC). We performed the genetic assessment of 637 patients with suspected HBOC. The region corresponding to the breakpoints for the 9–12 del *BRCA1* was amplified by polymerase chain reaction (PCR). An analysis of the clinical data of the carriers and non-carriers was done, searching for characteristics that correlated with the deletion. The 9–12 del *BRCA1* was detected in 5% of patients with suspected HBOC (30/637). In patients diagnosed with ovarian cancer, 13 of 30 were 9–12 del *BRCA1* carriers, which represents 43%. We found a significant association between the 9–12 del *BRCA1* carriers with triple negative breast cancer and high-grade papillary serous ovarian cancer. We concluded that the detection of the 9–12 del *BRCA1* is useful as a first molecular diagnostic strategy in the Mexican population. In particular, it shortens the gap in genetic assessment in patients with triple negative breast cancer and ovarian cancer.

## Introduction

The main genes associated with hereditary breast and ovarian cancer are *BRCA1* and *BRCA2*, tumour suppressor genes whose germline mutations confer a high risk for the development of breast cancer (45–65%), and ovarian cancer (11–40%) [1–4].

The majority of pathogenic mutations in *BRCA1/BRCA2* genes are point mutations (80%). In 10 to 15% there are large rearrangements, such as deletions or duplications of exons [5–7]. In both genes, more than 3,000 mutations have been reported [8–9]. Within these, mutations with a founder effect have been described in populations such as the Ashkenazi Jews, who are considered a genetically homogenous population [10–11]. Despite the heterogeneity of the ancestry of Latin American populations, mutations with a founder effect have also been described in Brazil (*BRCA1* 5382insC and *BRCA2* c.156_157insAlu) [12], and Colombia (*BRCA1* 3450del4, A1708E, and *BRCA2* 3034del4) [13]. In Mexico, a mutation with founder effect has been reported, the deletion of exons 9 to 12 of *BRCA1* (9–12 del *BRCA1*) [14], which represents up to 30% of the total of the mutations identified [15–16]. This mutation is common in patients with ovarian cancer, in which the overall frequency of mutations in *BRCA1/BRCA2* is 28%, which is mainly attributable to this founder effect [15].

The detection of founder mutations in specific populations can be considered as a cost-effective option, making molecular diagnosis more accessible, with the implications of diagnosis, treatment, prognosis, and prevention that this entails [17].

In 2014, the Society of Gynaecologic Oncology (SGO) recommended genetic counseling and molecular tests to all patients with ovarian cancer [18]. The possibility of personalising the treatment according to the mutational state, as occurs in the choice of treatments with platinum compounds and poly (ADP-ribose) polymerase 1 (PARP1) inhibitors in patients with ovarian cancer, has extended the recommendation to most international guidelines [19–20]. However, important barriers have been found to implement the recommendation worldwide. The Latin American region, in particular, faces limitations of access to molecular analysis due to its high cost [17,21].

In this study, we have presented the results of a targeted search for 9–12 del *BRCA1* in a group of 764 patients and relatives cared for at the first Hereditary Cancer Clinic in Mexico. We have provided an overview of the main clinical characteristics in correlation with 9–12 del *BRCA1*, highlighting the diagnosis of triple-negative breast cancer and high-grade papillary ovarian cancer. Likewise, a targeted search for a founder mutation might be the most cost-effective molecular diagnosis strategy in patients with suspected hereditary breast and ovarian cancer (HBOC) in Mexico.

## Materials and methods

### Patient recruitment

A total of 764 genomic DNA samples were included, for targeted detection of the deletion of exons 9 to 12 of *BRCA1*. Of the total DNA samples, 637 corresponded to oncological patients assessed at the Hereditary Cancer Clinic or the National Cancer Institute (Mexico), due to the suspicion of hereditary breast and ovarian cancer (HBOC). The clinical suspicion relies on the personal history (early age of onset; tumor phenotype; bilateral presentation), and family history (relatives with associated tumors; degree of relationship), according to criteria established in the Genetic/Familial High-Risk Assessment: Breast and Ovarian of the National Comprehensive Cancer Network (NCCN) guidelines, version 2.2017 (https://www.nccn.org/). All patients received genetic counselling and medical follow-up, by a certified medical geneticist.

We also included 127 relatives at risk to be carriers of 9–12 del *BRCA1*, for whom pre-symptomatic detection was performed. All family members received the corresponding pre- and post-test genetic counselling by a medical geneticist.

All patients and relatives gave their informed consent for inclusion before they participated in the study. The study was conducted in accordance with the Declaration of Helsinki, and Institutional Review Board (Comité de Ética en Investigación, Instituto Nacional de Cancerología, Mexico: 12 CEI0901411) approval was obtained (CEI/1036/16).

### DNA isolation

For all patients enrolled, 3 ml of peripheral blood were collected. Peripheral blood DNA was extracted with the Wizard® Genomic DNA Purification Kit (Promega), following manufacturer’s instructions. DNA concentration was quantified with NanoDrod 2000® (Thermo). Integrity and purity of nucleic acids was verified by agarose gel electrophoresis and spectrophotometry, respectively.

### Amplification polymerase chain reaction (PCR) of 9–12 del *BRCA1*

Region corresponding to the sites of rupture of the deletion was amplified (exons 9 to 12, *BRCA1*) by means of PCR. Weitzel et al., (2007) reported a similar method [22]. However, the assay design and the primers are different. Briefly, the fragments corresponding to the normal and mutated alleles were amplified with the oligonucleotides B01del9-12, B02del9-12 and B03del9-12. The PCR endpoint was performed in a GeneAmp PCR System 9700, (Applied Biosystems) in a volume of 30 μL, 150 ng of gDNA and a final concentration of 1X DreamTaq Buffer (Thermo Scientific), 0.25 mM of dATP, dTTP and dCTP, 0.2 mM of dGTP, (Thermo Scientific) with 0.63 mM of -N7-dGTP, 7-deaza-dGTP (SIGMA), 0.33 μM of each oligonucleotide and 0.06 U of DreamTaq DNA Polymerase (Thermo Scientific). The reaction conditions were: 1) 94°C 5min; 2) 94°C for 30 sec, 50°C for 30 sec, 72°C for 35 sec, 10 cycles; 3) 94°C for 30 sec, 53°C for 40 sec, 72°C for 40 sec, 25 cycles; 4) 72°C for 10 min; 5). Subsequently, electrophoresis was performed on 1.5% agarose gel and 0.5 X of Tris-borate-EDTA (ethylenediaminetetraacetic acid) buffer (ChemCruz). The presence of bands corresponding to the mutated allele in the amplified samples was verified (S1 Fig). A second PCR was performed with the mutated allele samples using only the oligonucleotides B01del9-12 and B03del9-12 with the same amplification conditions. The amplification was corroborated by electrophoresis in 1.5% agarose gel and 0.5 X of Tris-borate-EDTA buffer (ChemCruz). To enhance the reproducibility of our results, a detail laboratory protocol would be found at protocols.io: doi.org/10.17504/protocols.io.4t8gwrw.

### Sanger sequencing

The presence of 9–12 *BRCA1* deletion was corroborated through Sanger sequencing. A volume of 5 ul of the amplified product was purified with 2 μL of ExoSAP-IT PCR Product Cleanup (Affymetrix) under the conditions indicated by the manufacturer. The sequencing reaction was performed with a BigDye Terminator v3-1 Cycle Sequencing Kit (Applied Biosystems), as indicated by the manufacturer. Then, the product was labelled with a BigDye XTerminator Purification Kit (Thermo Fisher Scientific). It was analysed in 3500 Genetic Analysers (Applied Biosystems). The presence of the deletion was corroborated by aligning the sequence obtained with the sequence in the FASTA format of the *BRCA1* gene NG_005905.2 RefSeqGene (http://www.ncbi.nlm.nih.gov, NCBI GenBank) through the eBioX 1.6 Beta programme 1 Build 26/1.5.1).

### Statistical analysis

The statistical analysis was carried out using the IBM SPSS Statistics v.24 programme. After the analysis based on descriptive statistics, group comparison methods were used for the analysis of the clinical characteristics of patients carrying the 9–12 *BRCA1*, with respect to non-carriers. For this effect, two-sided Fisher’s exact test was performed. A "p" value of 0.05 was considered significant. Correction for multiple comparisons was performed with the Benjamini-Hochberg false discovery rate method.

## Results

In patients with suspected HBOC syndrome, 30 (5%) were identified as carriers of the 9–12 del *BRCA1* (Table 1).

**Table 1:** Clinical and familial features of cancer patients carriers of 9–12 del *BRCA1*.

SAMPLE ID	AGE AT CANCER DIAGNOSIS (YEARS)/GENDER	CANCER TYPE	TUMOR HISTOLOGICAL FEATURES	OTHER TUMOR FEATURES	FAMILIAL CANCER HISTORY	NUMBER OF FAMILY MEMBERS WITH CANCER	TYPE OF TUMORS IN FAMILY MEMBERS	DEGREES OF FAMILY MEMBERS WITH CANCER
**HC266-1**	61/Female	Ovarian cancer	High grade serous carcinoma		Yes	1	Breast cancer	2^nd^
**HC373-2**	60/Female	Bilateral breast cancer	Invasive ductal carcinoma (both)	Triple negative	Yes	1	Gastric cancer	1^st^
**HC321-1**	42/Female	Bilateral breast cancer	Invasive ductal carcinoma	Triple negative	Yes	1	Head and neck cancer (NS)	1^st^
**HC911-1**	52/Female	Ovarian cancer	High grade serous carcinoma		Yes	1	Breast cancer	1^st^
**HC221-1**	45/Female	Ovarian cancer	High grade serous carcinoma		Yes	1	Gastric cancer	1^st^
**HC352-1**	51/Female	Ovarian cancer	High grade serous carcinoma		Yes	1	Gynecologic cancer (NS)	1^st^
**HC175-1**	62/Female	Ovarian cancer	Poorly differentiated adenocarcinoma	NS	No	—	—	—
**HC487-1**	47/Female	Ovarian cancer	Serous carcinoma, poorly differentiated	NS	Limited information about family	—	—-	—-
**HC387-1**	52/Female	Ovarian cancer	Mixed tumor: high grade serous carcinoma and mucinous	Tumor size: Right ovarian: 1 cm. Left ovarian: 3.5 cm	Yes	2	Breast cancer; head and neck cancer (NS)	1^st^
**HC190-1**	48/Female	Ovarian cancer	Serous carcinoma, poorly differentiated	IHC: Ca-125 positive; WT-1 positive; mamma globin negative; vimentin negative	Yes	1	Colorectal cancer	1^st^
**HC13-1**	37/Female	Unilateral Breast cancer	Invasive ductal carcinoma	Triple negative	Yes	4	Unilateral breast cancer. Bilateral breast cancer. Liver cancer	1^st^ and 2^nd^
**HC16-1**	42/Female 47/Female	1^st^. Ovarian cancer 2^nd^. Unilateral breast cancer	1^st^. Poorly differentiated carcinoma 2^nd^. Invasive ductal carcinoma	2^nd^. ER positive, PR positive, Her2/neu negative; ki-67 30%	Yes	3	Ovarian cancer; breast cancer	1^st^ and 3^rd^
**HC35-1**	45/Female	Bilateral breast cancer	1^st^: Ductal carcinoma, triple negative. 2^nd^: In situ ductal carcinoma, triple negative	2^nd^ breast cancer IHC: p16(+); EGFR (+); CK 5/6 (+); CK 14 (+); Vimentine (+); p63 (-).	Yes	5	Breast cancer; prostate cancer; renal cancer	1^st^, 2^nd^ and 3^rd^
**HC36-1**	30/Female	Unilateral Breast cancer	Invasive ductal carcinoma	ER positive, PR positive, Her2/neu positive; ki-67 15%	Yes	5	Breast and ovarian cancer	1^st^ and 2^nd^
**HC109-1**	32/Female	Unilateral breast cancer	Invasive ductal carcinoma	Triple negative	Yes	3	Breast and pancreatic cancer	1^st^ and 2^nd^
**HC122-1**	43/Female	Unilateral breast cancer	Invasive ductal carcinoma	ER positive, PR positive and Her2/neu negative	Yes	2	Breast cancer	1^st^
**HC198-1**	35/Female	Unilateral breast cancer	Invasive ductal carcinoma	Triple negative	Yes	2	Breast, gastric cancer	2^nd^
**HC259-1**	34/Female	Unilateral breast cancer	Invasive ductal carcinoma	Triple negative	Yes	2	Breast, ovarian and gastric cancer	1^st^ and 2^nd^
**HC268-1**	30/Female	Ovarian cancer	High grade serous carcinoma		Yes	1	Breast cancer	1^st^
**HC659-1**	35/Female	Unilateral breast cancer	Invasive ductal carcinoma	Triple negative	Limited information about family	—	—	—
**HC773-1**	32/Female	Unilateral breast cancer	Pleomorphic lobular breast cancer	ER and PR positive, Her2/neu negative; ki-67 80%	Yes	2	Breast cancer; lymphoma non Hodgkin	1^st^ and 3^rd^
**HC816-1**	41/Female	Unilateral breast cancer	Invasive ductal carcinoma	Triple negative, ki-67 70%	No	—	—	—
**HC867-1**	47/Female	Ovarian cancer	High grade serous carcinoma	No	Yes	3	Breast and endometrial cancer	1^st^ and 2^nd^
**HC876-1**	36/Female	Bilateral breast cancer	1st: invasive ductal carcinoma; 2nd: Invasive ductal carcinoma	2^nd^: Triple negative; ki-67 90%	No			
**HC882-1**	58/Female	Bilateral breast cancer	Invasive ductal carcinoma (both)	Triple negative (both)	No			
**HC1030-1**	24/Female	Unilateral Breast cancer	Invasive ductal carcinoma	ER positive; PR negative; Her2/neu negative;	Yes	4	Breast cancer; skin (non-melanoma); gynecologic cancer (NS)	1^st^, 2^nd^ and 3^rd^.
**HC1251-1**	52/Female	Ovarian cancer	High grade serous carcinoma		Yes	2	Cervical cancer	1^st^ and 2^nd^
**HC1411-1**	31/Female	Unilateral Breast cancer	Invasive ductal carcinoma	Triple negative	Yes	1	Cervical cancer	2^nd^
**HC913-1**	34/Female	Bilateral breast cancer; Cervical cancer	Invasive ductal carcinoma (both)	2^nd^: Triple negative	No			
**HC86-1**	47/Female	Ovarian cancer	Mixed tumour		No			

NS, non specified; ER, estrogen receptor; PR, progesterone receptor; HER2/neu, human epidermal growth factor receptor 2; IHC, inmunohistochemistry; WT-1, Wilms tumor protein; EGFR, epidermal growth factor receptor; CK, creatine kinase.

As relevant clinical characteristics of HBOC carriers of the 9–12 del *BRCA1*, all were female. The mean age at the first cancer diagnosis was 43 years (SD 14.63), which did not have a significant difference with non-carrier patients.

In relation to oncological diagnoses, 18 patients had a history of breast cancer. In 12 of them, the condition was unilateral, while in 6 it was bilateral (2 synchronic, 4 metachronic). The histology of the tumors was predominantly of the infiltrating ductal carcinoma (23/24).

Regarding the tumour phenotype, in 7 of the patients with unilateral breast cancer and in all the patients with bilateral breast cancer, the tumors were triple negative. The remaining patients presented luminal A or B tumors.

In patients with ovarian cancer, 9–12 del *BRCA1* was detected in 43% (13/30) of the cases. All tumors had an epithelial component, highlighting high-grade serous epithelial tumours (10/13); the rest of the histologies corresponded to mixed tumours (3/13).

It should be noted that in two of the carriers, the presence of multiple cancer diagnoses were documented. In the first case, it was a patient with ovarian cancer and metachronous breast cancer. In the second case, the patient had a history of cervical cancer and metachronous bilateral breast cancer.

In relation to family history, 80% of the patients had cancer background, highlighting breast cancer in first- and second-degree relatives. In six of the carriers there was not family cancer history, so the suspicion of HBOC lies in age at diagnosis or tumor phenotype. There was not information available on the health status of their family in two of the patients.

In the case of the 127 family members included, they extended the information about their genealogy (S1 Table). Of them, 55 (43%) were identified as asymptomatic carriers of 9–12 del *BRCA1*. Of the patients identified as carriers, most of then were female (36/55).

In the pedigree, 100% of the relatives identified as asymptomatic carriers, had a positive history of at least one first- or second-degree relative with breast cancer. The average of cancer family members was three.

Additionally, the association between carry the 9–12 deletion *BRCA1* with different clinical features such as age at diagnosis, type of cancer, tumor phenotype, was analyzed among patients, carriers and non-carriers.

The triple negative tumour phenotype in breast cancer was statistically associated to 9–12 del *BRCA1* carriers, when compared with the group of non-carriers (73% versus 21%, p = 0.0005; two-sided Fisher's exact test). Also, an association was found with diagnosis of ovarian cancer, particularly high-grade papillary serous histology (p = 0.0004; two-sided Fisher's exact test). Both associations remain significant after adjustment for multiple comparisons by the Benjamini-Hochberg method.

## Discussion

The identification of patients with HBOC, allows the personalisation of oncological treatment and prognosis, as well as the establishment of risk reduction measures for cancer, both in patients and in family members, constituting the strategies of greater effectiveness in cancer prevention [23]. Therefore, it has become a daily practice in the multidisciplinary care of patients with breast and ovarian cancer [24].

Germline mutations of the *BRCA1* and *BRCA2* genes are the main etiological component of HBOC [1–4]. Genomic large rearrangements (LGRs), such as exon deletions or duplications, are defined as those alterations that involve more than 500 kb of DNA [25]. For the identification of LGRs, the most widely used technique is the Multiplex Ligation Probe Assay (MPLA) [26].

So far, more than 130 LGRs have been identified in the BRCA genes [27]. Most of the LGRs are in *BRCA1*, due to the large amount of Alu repeats that comprise it, which makes it susceptible to errors in recombination. In populations where founder effects exist, the first molecular diagnosis consists of the directed search for it as the first option due to its frequency. This strategy allows the optimisation of time and economic resources [17, 21].

Large rearrangements, such as founder mutations in the BRCA genes, have been reported predominantly in the Caucasian population (Germany, Greece, Spain) [27–29]. In the Dutch population, two large genomic rearrangements, the deletion of 3.8 kb of exon 13, and the deletion of 510 bp of exon 22, represent approximately 25% of the mutations identified in the cases of hereditary breast cancer [30]. In Latin America, the 9–12 del *BRCA1* represents the first large genomic rearrangement with a founder effect in the region [7].

Weitzel et al. identified 9–12 del *BRCA1* for the first time in 2005, studying a population at risk of hereditary cancer of Latin American origin (predominantly of Mexican ancestry) residing in the United States of North America [15]. In 2007, the same group of researchers reported the molecular characterisation of the deletion [22]. A later survey in the Colombian population documented its absence by looking for it in a targeted way in 538 patients with suspected HBOC [31]. The above data is considered part of the evidence of the founder characteristic in the Mexican population.

A year later, in 2013, by genotyping carriers for 9–12 del *BRCA1* with Mexican ancestry and residents predominantly in the southern United States of North America, it was estimated that the mutation originated 74 generations ago or 1,480 years earlier [32].

Until that date, a limitation of the studies that involved the diagnosis of 9–12 del *BRCA1* was due to the absence of data in a purely Mexican population. Therefore, in 2014, Torres-Mejia et al., performed a molecular analysis of recurrent mutations in *BRCA1/BRCA2* in 810 unselected breast cancer patients, residents of three Mexican cities (Mexico City, Veracruz and Monterrey). The 9–12 del *BRCA1* was the most common mutation, representing 22% of the mutations identified [33].

Two other publications with projects conducted in the Mexican population reported relevant data regarding 9–12 del *BRCA1*. In the first study, which involved patients with breast and ovarian cancer, without suspicion of hereditary cancer, 9–12 del *BRCA1* represented 33% of the total mutations identified, 35% in the subgroup of patients with ovarian cancer and 29% in that of patients with breast cancer, constituting the most frequent mutation [15]. In the second study, whose objective was the molecular analysis of the BRCA genes in 190 patients with triple negative breast cancer diagnosed before the age of 50 years, the 9–12 of the *BRCA1* accounted for 41% of mutations identified [16].

Recently, the world's largest study of prevalence of *BRCA1* and *BRCA2* mutations has been published. This study included 29,700 *BRCA1/BRCA2* mutation carriers from 49 countries, distributed across 6 continents. In that report, the 9–12 del *BRCA1* is the most frequent mutation in Mexico, and the fourth most prevalent in Hispanic/Latino ethnicity [7].

Our results show a 5% frequency of 9–12 del *BRCA1* when its directed search is applied as the first molecular diagnostic strategy to a group of 637 patients with clinical suspicion of HBOC.

Previous studies of the Mexican population show that 9–12 del *BRCA1* represents between 23 and 41% of the total number of mutations in *BRCA1* (Table 2.).

**Table 2:** Frequency of *BRCA1* mutations in Mexican population: Proportion representing 9–12 del *BRCA1*.

Study/Author	Frequency (%) of *BRCA1* point mutations	Frequency (%) of 9–12 del *BRCA1*	Other LGRs identified
**190 women with TN breast cancer/Villarreal-Garza C, et al., 2015 [16].**	58	41	Not searched
**188 women (92 with ovarian cancer and 96 with breast cancer)/Villarreal-Garza C, et al., 2015 [15].**	47	38	15
**300 women with HBOC suspected/Quezada-Urban R, et al., 2018 [34].**	69	31	Not searched
**195 women (101 with HBOC; 22 with sporadic breast cancer; and 72 healthy women)/Zayas-Villanueva OA, et al., 2019 [35].**	38.5	23	38.5
**Current study data, 687 women with HBOC suspected**	69	31	Not searched

LGRs = Genomic large rearrangements. TN = Triple negative breast cancer tumors. HBOC = Hereditary Breast and Ovarian Cancer.

In our study, the 9–12 del *BRCA1* represents the 30.92%, given that 67 point mutations were identified in *BRCA1* by sequencing (data not shown). It should be noted that if the study population is stratified by characteristics, such as the triple negative phenotype, the proportion that represents 9–12 del of the total *BRCA1* mutations is higher.

Implementing the targeted detection as a first step represents obtaining an outcome by an accessible, efficient and effective technique, in economic terms (less than $10), and in waiting time (less than 1 week). Thus, the subgroup of patients identified as carriers no longer required other testing, such as sequencing/MLPA, which is helpful due to the challenges of accessibility that had Latin America [17,36,37]. In this context in Mexico, the cost of a cancer susceptibility genes molecular test ranges between $ 500 and $ 5,000, according to different commercial laboratories. A Mexican family receives an average of 88 pesos per day (less than $ 5), evidencing the accessibility problem that we face [38]. Having a cost-effective strategy for a founder mutation related to HBOC (with a cost less than $ 10), could shorten that gap, and even make it accessible to be cover by the public health system. Additionally, it was possible to benefit 127 asymptomatic individuals, identifying 43% as carriers in the context of a predictive study.

Because LGRs have been considered particularly penetrating mutations, being associated with greater cancer risks, and even certain "high-risk characteristics" [39], we intentionally sought their correlation with the clinical data present in the population studied. In this way, the correlation of carriers of 9–12 del *BRCA1* with triple negative breast cancer was found, as well as high-grade papillary serous ovarian cancer. These data are in congruence with previous data reported in our institution since the 9–12 del *BRCA1* was found in 9% of 190 patients with triple negative breast cancer, under 50 years of age, not selected for their family history [16]. Similarly, the 9–12 del *BRCA1* was identified in 9% of 92 unrelated patients with ovarian cancer of different histologies [15].

**Fig 1:**
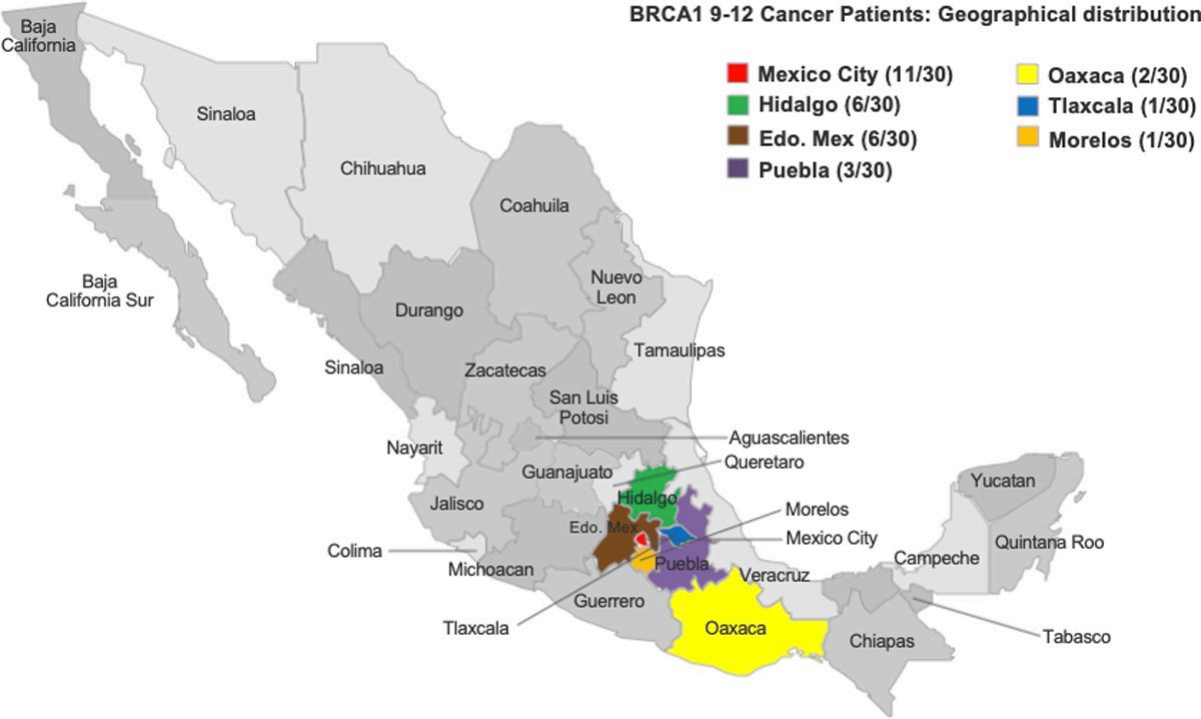
Geographic distribution (place of birth) of the carriers of the 9–12 del *BRCA1*.

For this same reason, one of the main limitations of our study is that it mainly reflects the population of the centre of the country (Fig 1). Despite being the national cancer institution, the territorial extension of the country (1,964 million km²) and other migratory, social and cultural phenomena, limit the generalisation that can be made of the distribution of the founder mutation 9–12 del *BRCA1* in our population. More studies will be needed from other regions of the country to determine whether their behaviour remains constant or has considerable variation.

**Fig 2:**
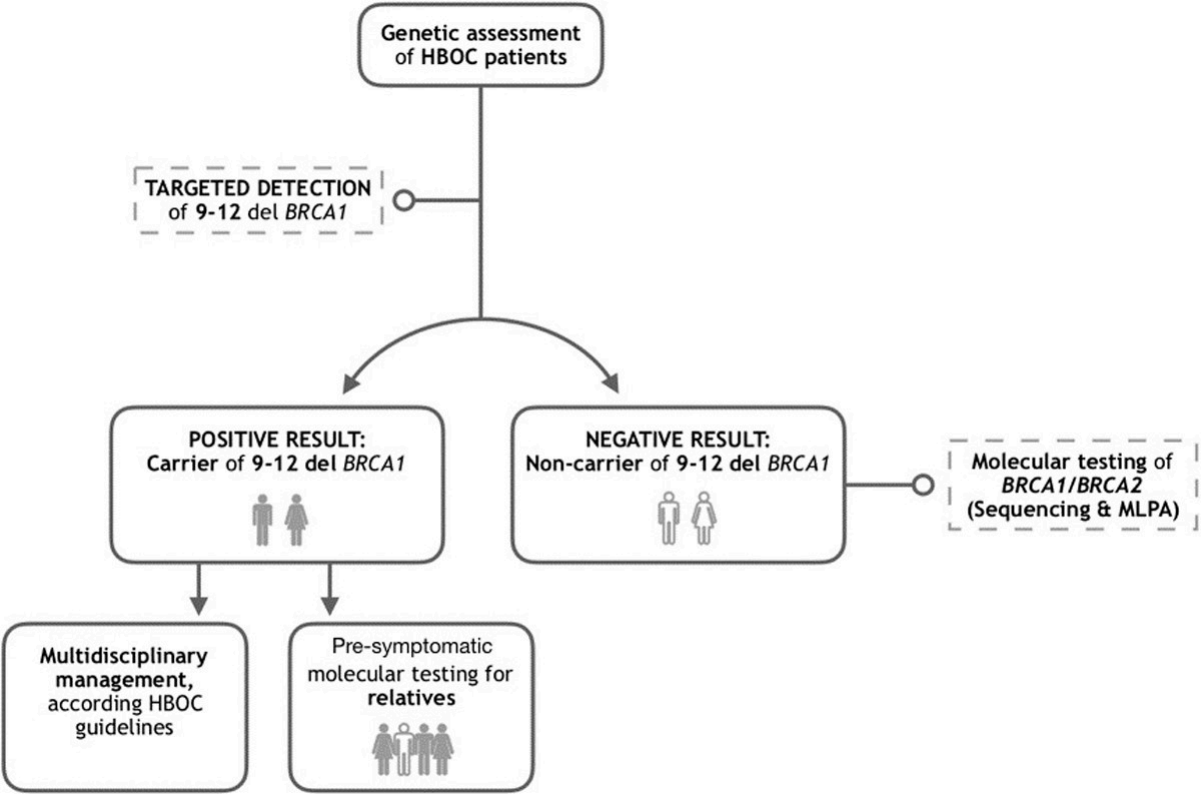
Proposed approach for Mexican hereditary breast and ovarian cancer patients.

However, one of our contributions lies in the possibility of making molecular testing more accessible to our population, considering as a first line of diagnosis the detection for 9–12 del *BRCA1* in all patients with suspected HBOC (Fig 2). In this way, we propose that all patients with suspected HBOC, who were born in the central region of our country, begin their diagnostic approach with the targeted detection of 9–12 del *BRCA1*. If the result is negative, it will be possible to continue with the sequencing and search for large rearrangements (by MLPA, for example) in the associated susceptibility genes.

We emphasise the possibility of its finding in patients with high-grade papillary serous ovarian cancer and triple negative breast cancer. Particularly in ovarian cancer and in accordance with international recommendations for universal genetics assessment, our proposal guarantees access to a first molecular screening line, which is potentially feasible throughout the country. Likewise, it allows the identification of those patients who may be susceptible to a targeted treatment, such as PARP1 inhibitors, which currently have national approval for the treatment of recurrent ovarian cancer.

## Supporting information

S1 Fig Targeted detection for the 9–12 del *BRCA1*. (TIF)

S1 Table Familial features of relatives tested for pre-symptomatic diagnosis of 9–12 del *BRCA1*. (PDF)
