# Supplementary figures and images for: Mexican BRCA1 founder mutation: Shortening the gap in genetic assessment for hereditary breast and ovarian cancer patients
Source: PLoS One. 2019 Sep 23;14(9):e0222709. doi: 10.1371/journal.pone.0222709 (PMC6756553; doi:10.1371/journal.pone.0222709)

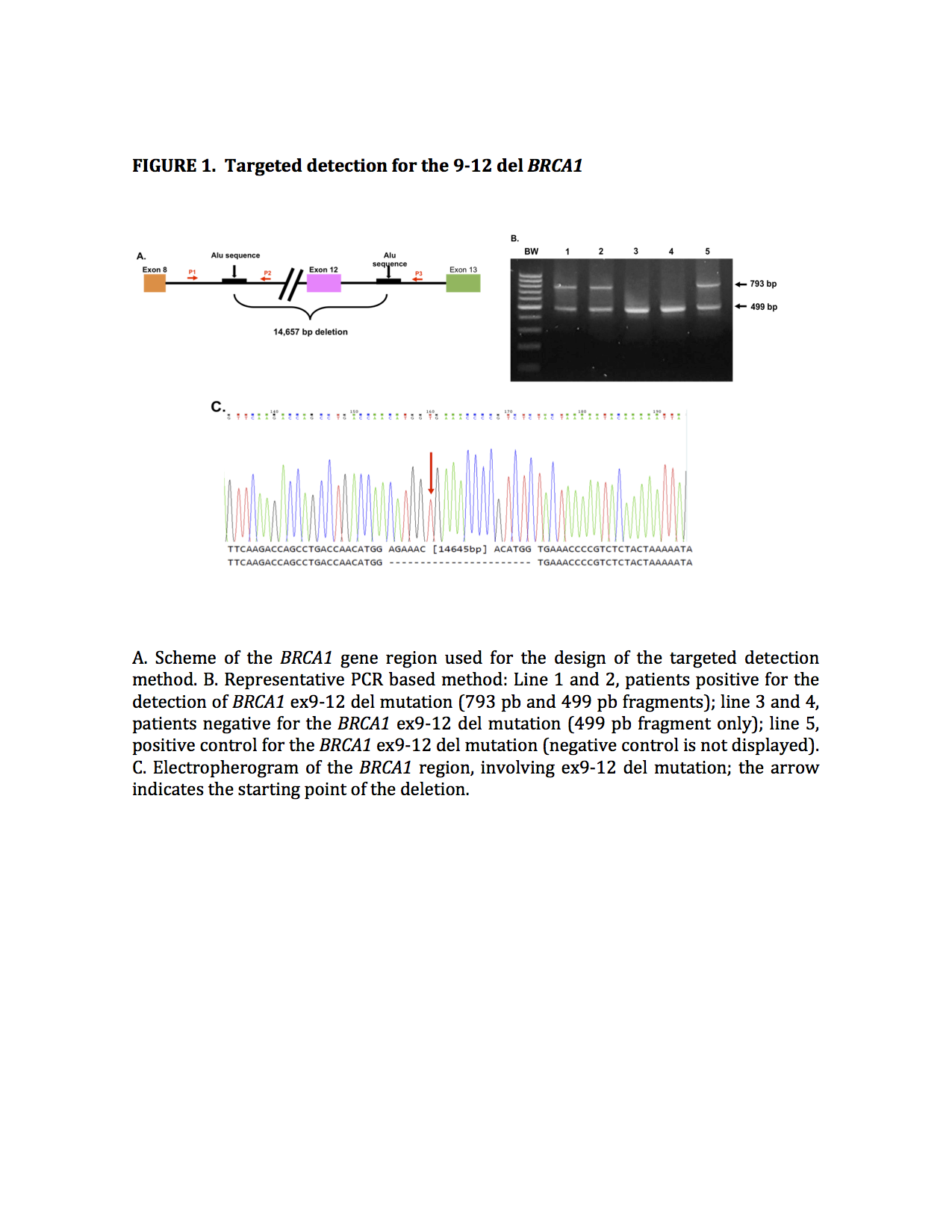

Supplement: S1 Fig — (TIF) [file pone.0222709.s001.tif]
