# Supplementary material for: Mexican BRCA1 founder mutation: Shortening the gap in genetic assessment for hereditary breast and ovarian cancer patients
Source: PLoS One. 2019 Sep 23;14(9):e0222709. doi: 10.1371/journal.pone.0222709 (PMC6756553; doi:10.1371/journal.pone.0222709)
Supplement: S1 Table — (PDF) [file pone.0222709.s002.pdf]

S1 TABLE. Familial features of relatives tested for pre-symptomactic diagnosis of 9-12 del *BRCA1*

FAMILY ID	NUMBER OF FAMILY MEMBERS TESTED FOR DEL 9-12 *BRCA1*	GENDER OF FAMILY MEMBERS TESTE FOR DEL 9-12 BRCA1	TYPE OF TUMORS IN THE FAMILY	NUMBER OF FAMILY MEMBERS AFFECTED BY CANCER	DEGREE OF KINSHIP (RELATIVES WITH CANCER)
F1	5	5 females	Information not available	1	2º
F2	3	3 females	Breast; cervical; bronchogenic.	3	1st
F3	4	4 females	Breast; prostate; cervical; colorectal cancer	4	1st and 2º
F4	2	2 females	Breast cancer	2	1st
F5	8	4 females; 4 males	Breast; ovarian; prostate; esophageal; hepatic; renal; testicultar	13	1st, 2º, 3º
F6	1	1 female	Information not available	---------	----------
F7	8	6 females; 2 male	Breast	3	1st
F8	2	2 females	Breast; endometrium	8	1st, 2º
F9	17	13 females; 4 males	Cervical; stomach; renal	3	1st; 2º
F10	1	1 female	Breast; cervical; duodenum	5	1st; 2º; 3º
F11	2	2 females	Breast; leukemia	3	1st; 2º; 3º
F12	7	4 females; 3 males	Melanoma; Cancer type not specified	2	1st; 2º
F13	7	5 females; 2 males	Breast	1	1st
F14	4	1 female; 3 males	Breast; lung; stomach	3	1st; 2º
F15	8	7 females; 1 male	Breast	2	1st
F16	2	2 males	Breast; Hodgkin's lymphoma	2	3º; 4º
F17	5	2 females; 3 males	Breast; stomach	2	2º
F18	3	2 females; 1 male	Ovarian	1	1st

F19	6	4 females; 2 males	Non family history	------	-------
F20	4	2 females; 2 males	Non family history	-----	------
F21	1	1 female	Non family history	-----	-----
F22	5	2 female; 3 males	Hepatic; cervical	2	2º
F23	11	6 females; 5 males	Ovarian; breast	2	1st; 2º
F24	2	1 female; 1 male	Breast; endometrium; bile ducts; prostate	5	1st; 2º
F25	3	2 females; 1 male	Renal; stomach	2	1st; 2º
F26	2	2 females	Breast; ovarian; stomach; lung	7	1st; 2º; 3º
F27	1	1 female	Breast	4	4º; 5º
F28	1	1 female	Breast	3	1st; 2º
F29	1	1 female	Colorectal	1	2º
F30	1	1 female	Breast; ovarian; stomach	5	1st; 2º ; 3º
